# Citrus auraptene suppresses cyclin D1 and significantly delays N-methyl nitrosourea induced mammary carcinogenesis in female Sprague-Dawley rats

**DOI:** 10.1186/1471-2407-9-259

**Published:** 2009-07-29

**Authors:** Prasad Krishnan, Karen J Yan, David Windler, Jesse Tubbs, Robert Grand, Benjamin DL Li, C Marcelo Aldaz, Jerry McLarty, Heather E Kleiner-Hancock

**Affiliations:** 1 Department of Pharmacology, Toxicology and Neuroscience, LSUHSC-Shreveport, Louisiana, USA; 2 Breast Cancer Focus Group, Feist-Weiller Cancer Center, Shreveport, Louisiana, USA; 3 Department of Surgical Oncology, LSUHSC-Shreveport, Louisiana, USA; 4 Department of Carcinogenesis, U.T.M.D. Anderson Cancer Center, Smithville, Texas, USA; 5 Department of Medicine, LSUHSC-Shreveport, Louisiana, USA

## Abstract

**Background:**

Breast cancer is a major problem in the United States leading to tens of thousands of deaths each year. Although citrus auraptene suppresses cancer in numerous rodent models, its role in breast cancer prevention previously has not been reported. Thus, our goal was to determine the anticarcinogenic effects of auraptene against breast cancer.

**Methods:**

The effects of auraptene on cell proliferation of MCF-7 and MDA-MB-231 human breast carcinoma cells in culture was assessed by measuring metabolism of a substrate to a formazan dye. Dietary effects of auraptene on tumor incidence, multiplicity and latency were studied in the N-methyl nitrosourea (MNU) induced mammary carcinogenesis model in female Sprague Dawley rats. The concentration of auraptene in rat tissues was analyzed by reverse phase HPLC. Cyclin D1 expression in MCF-7 cells and rat tumors was measured by western blot.

**Results:**

Auraptene (500 ppm) significantly delayed median time to tumor by 39 days compared to the MNU only group (p < 0.05, n = 24–26). Auraptene (10 μM) reduced Insulin like Growth Factor-1 (IGF-1, 10 ng/mL)-induced cyclin D1 expression by 40% in MCF-7 cells. In comparison, western blot analysis of rat mammary tumors (n = 10 per group) confirmed that auraptene (500 ppm) significantly reduced (p < 0.05) cyclin D1 expression by 49% compared to the MNU only group. Analysis of rat mammary tissue extract by HPLC with fluorescence detection indicated an average concentration (means ± S.E.) of 1.4 ± 0.5 μM and 1.8 ± 0.3 μM in the normal mammary glands of the auraptene 200 ppm and 500 ppm groups, respectively. The concentration (means ± S.E.) of auraptene in the mammary tumors of the auraptene 200 ppm group was 0.31 ± 0.98 μM.

**Conclusion:**

Overall, these observations suggest that the predominant effect of auraptene was to delay the development of tumors possibly through the suppression of cyclin D1 expression. These results point to the potential chemopreventive effects of auraptene in mammary carcinogenesis.

## Background

Breast cancer is a major cause of death in women under the age of 55. In the year 2008, 184,450 new cases of breast cancer (182,460 women and 1990 men) and 40,930 deaths (40,480 women and 450 men) are expected [1]. Breast cancer is second only to lung cancer as the leading cause of cancer deaths in women [1]. Many risk factors have been attributed to breast cancer occurrence. Genetic susceptibility accounts for only ~10% of human breast cancer [1]. Known environmental risk factors include radiation, obesity, and alcohol use [1].

Since breast cancer remains as a major threat to women's health all over the globe, prevention of breast cancer is an ideal strategy. Generally carcinogenesis is considered to consist of three steps- initiation, promotion and progression [2]. The promotion stage is usually a lengthy process which could be reversible. Thus there is an opportunity to prevent carcinogenesis from progressing to the malignant stage [2]. Many natural dietary compounds are being tried for chemoprevention of cancer One such agent, auraptene, is a geranyloxy-coumarin obtained from citrus fruits [3]. Recently it has been shown that the leaves of an aromatic plant Zanthoxylum schinifolium (used in Korea and Japan as a food flavor and herbal medicine) [4] and fruits of Paliurus ramosissimus [5] also contain auraptene. Auraptene was effective in preventing the chemical carcinogenesis in various rodent models including skin [3], tongue [6], esophagus [7], liver [8,9], and colon carcinogenesis [10-13]. Auraptene has also been shown to have protective effects in a prostate [14] cancer model. However, its chemopreventive effects have not been addressed in animal models of breast cancer. Several mechanisms have been reported for auraptene's chemopreventive properties including induction of carcinogen detoxifying enzymes, induction of apoptosis [3,4,15,16], inhibition of free radical generation [17] metalloproteinase [18,19], inflammatory pathways [6,20] and polyamine synthesis [6,20]. These varied mechanisms and protective effects in rodent models of cancer combined with the low risk of toxicity [21] suggest that auraptene might be a good candidate for breast cancer chemoprevention. In addition, coumarins generally possess high oral bioavailability [22]. Studies from our laboratory have also suggested that orally administered citrus coumarins were absorbed and distributed to various organs in the body [15,23,24]. Based on these studies, we hypothesized that auraptene could suppress mammary carcinogenesis.

To address whether auraptene may be effective against breast cancer, the effects of auraptene on the proliferation of human breast cancer cells, MCF-7 and MDA-MB-231, were assessed. Next, the effect of auraptene on protein expression of the cell cycle protein, cyclin D1 was analyzed. We hypothesized that auraptene could suppress mammary carcinogenesis by suppressing cyclin D1 expression. The rat mammary MNU model is a well-established model for the evaluation of the chemopreventive activities of drugs and natural products [25]. Rats treated with the carcinogen MNU will develop mammary adenocarcinomas as early as 4 weeks post treatment and achieve nearly 100% tumor incidence by 20 weeks[25]. About 50% of the tumors that arise possess a mutated *ras*. The histopathology is considered to be similar to humans in that most of the tumors are adenocarcinomas. MNU is an alkylating agent that does not require metabolic bioactivation to exert its carcinogenic effects [25]. Following the *in vitro *studies, the chemopreventive effects of auraptene were assessed in the MNU-induced rat mammary carcinogenesis model and the expression of cyclin D1 in mammary tumors was analyzed by western blot. Since auraptene is intended to be part of the diet, the auraptene concentration in the mammary glands was measured. In addition, auraptene concentration in mammary tumors and livers of the rats were also measured.

## Methods

### Chemicals

Auraptene was purchased from LKT Laboratories Inc., (St. Paul, MN). The powdered diet (Teklad- 7001, 4% mouse/rat diet) was obtained from Harlan Teklad, (Madison, WI). MNU (N1517) was purchased from Sigma, (St. Louis, MO). The O.C.T. compound (#4583) was obtained from Tissue-Tek, (Torrance, CA).

### Cell Proliferation Assay

The human breast carcinoma cell line MDA-MB-231 was obtained from ATCC (Manassas, VA) and grown in a sterile humidified environment at 37°C and 5% CO_2_: 95% air with complete DMEM media. The proliferation study was done by MTT assay which uses a tetrazolium compound [3-(4, 5-dimethylthiazol-2-yl)-2,5-diphenyltetrazolium bromide; MTT], which is converted into a formazan product by living cells. The MDA-MB-231 cells were plated in a 96 well plate at 12,500 cells/well at a confluency of 40%. The serum containing media was replaced with serum free media 5 hrs before treatment. The cells were then treated with auraptene at 1 – 50 μM auraptene in DMSO (0.1%, v/v). The control cells received vehicle only (DMSO, 0.1% v/v). After 24 hrs of treatment the cells were treated with MTT for 2 h and the absorbance was measured at 540 nm.

The human breast carcinoma cell line MCF-7 was obtained from ATCC (Manassas, VA) and grown in a sterile humidified environment at 37°C and 5% CO_2_: 95% air with complete DMEM media. The MCF-7 cells were plated in a 96 well plate at 30,000 cells/well at a confluency of 40%. The cells were serum starved for 24 h. At 22 h of serum starvation, the cells were treated with auraptene at 1 – 50 μM auraptene in DMSO (0.1%, v/v). The control cells received vehicle only (DMSO, 0.1% v/v). At 24 h of serum starvation, the cells were treated with IGF-1(10 ng/mL). After 24 hrs of IGF-1 treatment, the cells were treated with MTS [3-(4,5-dimethylthiazol-2-yl)-5-(3-carboxy methoxy phenyl)-2-(4-sulfophenyl)-2H-tetrazolium], for 2 h and the absorbance was measured at 490 nm.

### Animals

A total of 128 female Sprague-Dawley rats (28 days of age), purchased from Harlan Teklad (Madison, WI), were used for this experiment. They were housed two per cage, in a temperature and humidity controlled AAALAC facility under 12 h light/dark cycle. All procedures were approved by LSUHSC Institutional Animal Care and Use Committee in accordance with NIH guidelines. They were fed test diets as described below and allowed access to food and water *ad libitum*.

### Treatment of rats

**Figure 1:**
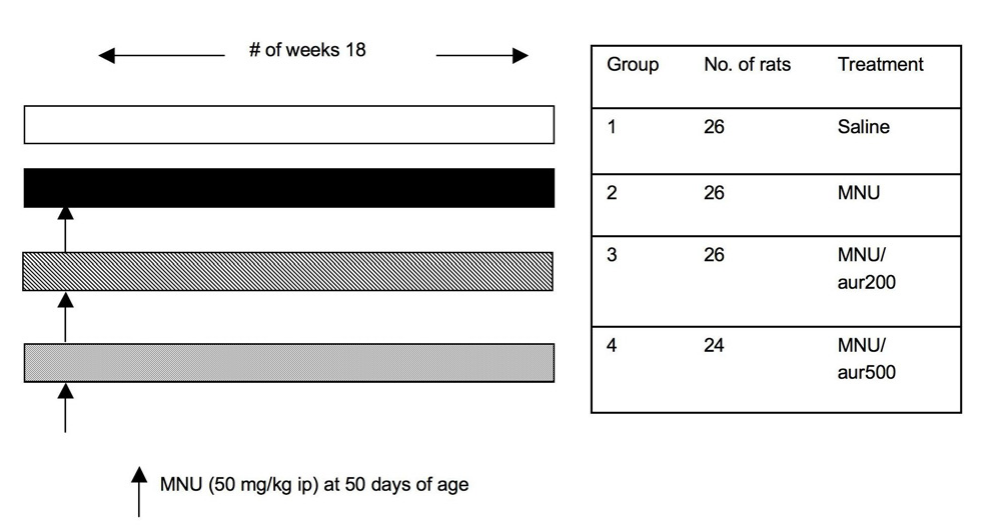
**Experimental design of the rat mammary carcinogenesis study**.

Rats (28 days of age) were divided up randomly into groups of 24–26 each and were handled daily to get them accustomed to human touch. At 42 days of age the rats were shifted to the test diet. Control group and MNU only group were fed with Teklad (powdered diet) only. MNU/Auraptene 200 ppm group and MNU/Auraptene 500 ppm group received 200 ppm and 500 ppm of auraptene respectively, mixed in the diet for the duration of the study. The doses were selected from the pilot study conducted with a small number of animals. In addition to the 200 ppm dose of auraptene, which indicated the chemopreventive effect of auraptene, a higher dose of auraptene at 500 ppm was added to this study to see any increased chemopreventive effects. Auraptene has been administered in diet at 100 ppm and 500 ppm in other models of cancer [6,9,13]. The diet was prepared once a week and stored in cold room. The food was replaced on Monday, Wednesday and Friday or as needed. The stability of auraptene was tested by HPLC analysis and the results showed that auraptene was not degraded in the animal room conditions (data not shown). At 50 days of age all the groups except the control group received intraperitoneal MNU injection (50 mg/kg bw in sterile acidified saline). The control group received vehicle only. The scheme is shown in Figure 1. The rats were palpated for tumors twice a week starting at 2 weeks after MNU administration. Any tumors felt during palpation were recorded by location. The rats were weighed once a week. A diet consumption analysis was done once to see if rats were consuming enough feed across all the groups and there was no significant difference in food consumption between the groups (data not shown). Any rats found to be moribund during the study were sacrificed within 24 h and necropsied. At 18 weeks after MNU administration, rats were euthanized by carbon dioxide asphyxiation and necropsied. The tumor location and weight of the tumor were marked in the necropsy sheet. Mammary fat pads and the tumors were collected in formalin, OCT compound and liquid nitrogen.

### Tissue Extraction and HPLC-Fluorescence analysis

The concentration of auraptene was measured in mammary glands, livers and tumors of rats. The specimens from the animals were weighed and minced on a glass plate kept on ice. The minced sample was added to 0.05 M Tris buffer, pH 7.4, containing 0.25 M sucrose in 1 ml. To 1 ml of this homogenized sample, 80 μL of 100 μM curcumin was added as internal standard followed by vortexing. The sample was then extracted twice with ethyl acetate: acetone (2:1 v/v). The combined supernatant was then dried under N_2 _gas, resuspended in 1 mL 70% methanol/H_2_O and analyzed by HPLC (Shimadzu, SIL-10AF) with UV (254 nm) and fluorescence detection (Shimadzu, RF-535) at excitation and emission wavelengths of 329 nm and 390 nm respectively. The flow rate in HPLC was 1 ml/min with C18*5μ column. The mobile phase started with 80% methanol in water up to 20 minutes and was increased in a linear gradient up to 100% methanol over the next 5 minutes. Under these conditions, auraptene eluted at 14 minutes and curcumin at 6 minutes. The ratio of area under the curve for auraptene at different concentrations to that of curcumin was plotted to make the standard curve. The concentration of auraptene in the specimens was calculated from the standard curve. For the calculation of concentration, 1 gm of the tissue was assumed to occupy a volume of 1 ml.

### Histopathology

The tumors were histopathologically analyzed to confirm the malignancy of the tumors palpated during the study. For the histopathological analysis, 4 μm thick sections were cut and then rehydrated by xylene and graded ethanol. The specimens were then stained with Haematoxylin and Eosin. The H&E stained specimens were examined by a pathologist to histopathologically classify the tumors as described by Russo J et al[26]. The tumors were broadly classified into two- adenocarcinoma (malignant) and benign tumors. The benign epithelial neoplasms in this animal model include papillary, tubular and lactating adenoma. The malignant epithelial neoplasms include ductal carcinoma (invasive and non-invasive) with cribriform, comedo, solid and papillary pattern. These benign and malignant tumors in the rats have corresponding human lesions [26].

### Western blot analysis

#### MCF-7 cells

To study the effect of auraptene on the expression on cyclin D1, MCF-7 cells were plated in eight 12-well plates at 1.5 × 10^4 ^cells/well. The cells were incubated at 37°C till the next day in media with serum. The cells were serum starved for synchronization in serum free media for the next 24 h. At 22 h of serum starvation, the cells except in DMSO only and IGF-1 only groups, were treated with 10 μM auraptene in DMSO. At 24 h of serum starvation, the cells except in DMSO group were treated with 10 ng/mL of IGF-1. The cells were harvested at 5,15 and 30 minutes and at 1 h, 2 h, 4 h, 8 h and 24 h after IGF-1 treatment by directly adding boiling Laemmeli buffer. The cyclin D1 expression was then analyzed by western blot. In short, equal volumes (15 μL) of the sample in Laemmeli buffer was loaded into each well and resolved. The separated proteins were then electroblotted on a 0.45 μm pore size PVDF membrane (Immobilon-P, Millipore, Billerica, MA). After the protein transfer the membrane was placed in Millipore SNAP id Protein Detection System. The membranes were blocked in 0.5% milk in TBS-T buffer by pouring the blocking buffer through the system connected to vacuum. Primary incubation of the membranes was carried out using 1:500 dilution of polyclonal rabbit anti-cyclin D1 antibody (#2922; Cell Signaling Technology, Danvers, MA). Secondary incubation of the membrane was then carried out by using a 1:1000 dilution of anti-rabbit antibody. The bands were developed by detection using Pierce ECL western blot substrate kit (# 32209; Pierce, Rockford, IL). The blots were scanned using the Epson system (# 1650; Epson America, Long Beach, CA). The band intensity was evaluated using the Image-J software. The cyclin D1 expression was normalized to β-actin.

#### Rat mammary tumors

The mammary tumors from the female Sprague-Dawley rats were analyzed for cyclin D1 expression and eukaryotic Initiation Factor 4E (eIF4E) as previously described [27]. Briefly, samples were first homogenized in RIPA buffer [50 mM Tris-HCL (pH 7.4), 1% Triton-X, 150 mM NaCl, 1 mM EDTA, 1 mM sodium orthovanadate, 1 mM PMSF, 1 μg of aprotinin, leupeptin and pepstatin and 1 mM sodium fluoride]. The proteins were measured by Bradford method [28] (using BSA as a standard), followed by the addition of 2 × sample buffer [0.125 M Tris, 10% (w/v) SDS, 40% (v/v) glycerol, 0.04% (w/v) bromophenol blue, and 80 mM dithiothreitol]. Samples were heated to 95°C for 5 min, and 10 μg of protein/well was loaded and resolved. The proteins were then transferred and detected for cyclin D1 expression as mentioned above. Lysate from MCF-7 cells treated with IGF-1 was used as positive control for cyclin D1.

### Statistical Methods

The rat body weight data was compared between groups by One-Way ANOVA followed by Tukey test. Groups were compared for the total number of tumors per rat by the nonparametric Kruskal-Wallis test. Time to tumor was estimated and compared among groups using survival curve analysis by the Log-rank test. The Cochran-Armitage test was used to test for trends in binomial response to different dose levels. Analysis was performed using StatXact (Cytel Software Corporation, Cambridge MA) and SPSS (SPSS Inc., Chicago IL). The cell proliferation assays and western blot data were analyzed by One-Way ANOVA followed by Tukey test.

## Results

### Auraptene suppressed human breast cancer cell proliferation

**Figure 2:**
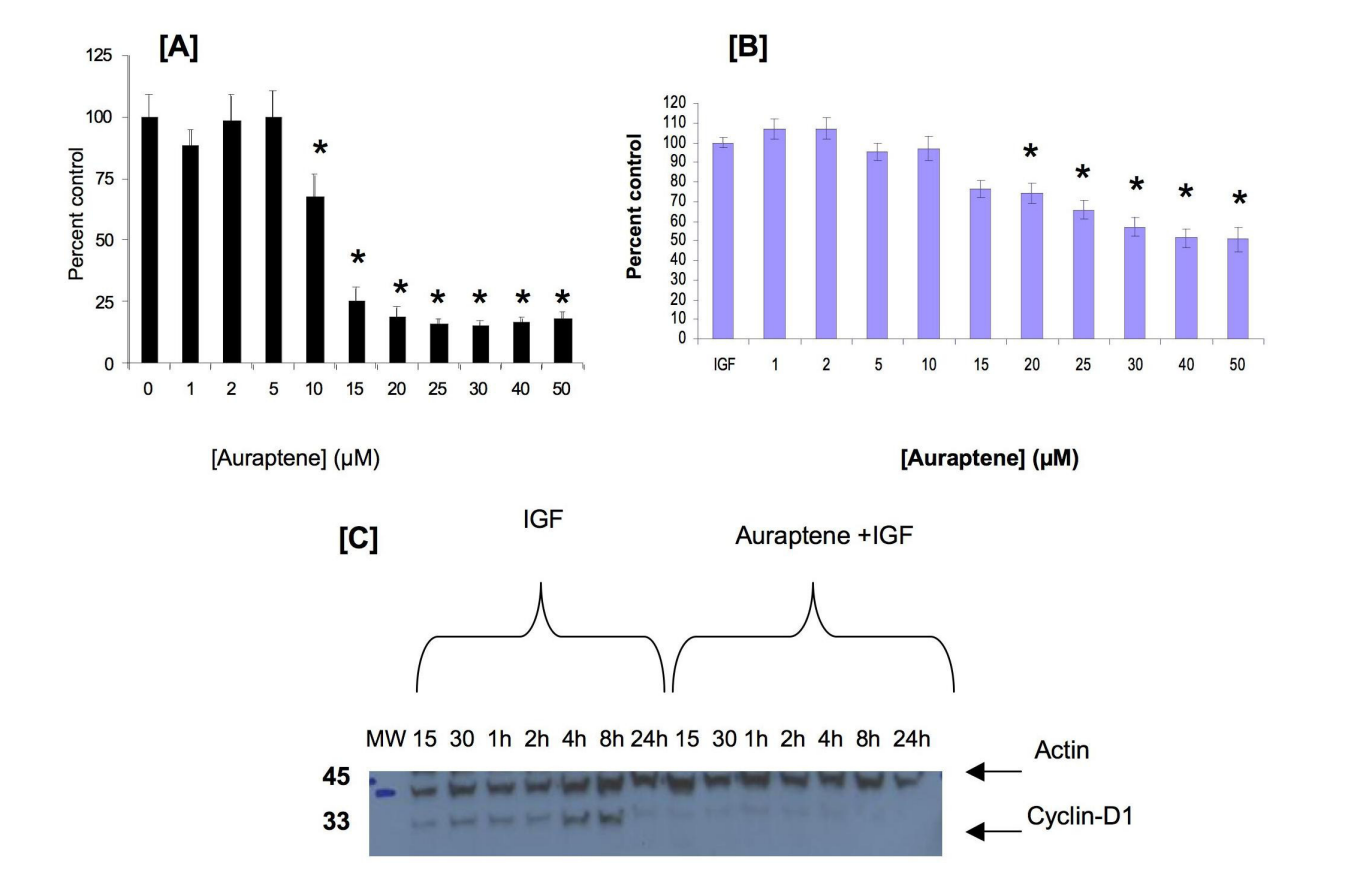
**Effects of auraptene on human breast carcinoma cells**. **(A) **Effects of auraptene on MDA-MB-231 cell proliferation. Cells were serum starved for 5 h and then treated with 1–50 μM auraptene in 0.01% DMSO. Cell proliferation was assessed using the MTT reagent. Figures represent % vehicle control (means ± S.E.) # Replicates = 2 plates at 6 wells each for the treatment groups and 12 wells for the control group (* statistically different from control group, p < 0.05, One-way ANOVA, Tukey test). **(B) **Effects of auraptene on MCF-7 cell proliferation. Cells were serum starved for 24 h. At 22 h after serum starvation the cells were treated with 1–50 μM auraptene in 0.01% DMSO. At 24 h serum starvation, the cells were treated with IGF-1 (10 ng/mL). Cell proliferation was assessed using the MTS reagent. Figures represent % vehicle control (mean± S.E.) # Replicates = 2 plates at 6 wells each for the treatment groups. (* statistically different from IGF-1 group, p < 0.05, One-way ANOVA, Tukey test). **(C) **Effects of auraptene on IGF-1 induced cyclin D1 expression. MCF-7 cells were serum starved for 24 h. At 22 h after serum starvation the cells were treated with10 μM auraptene in 0.01% DMSO. At 24 h serum starvation the cells were treated with IGF-1 (10 ng/mL). The cells were harvested at the 15, 30 minutes and at 1 h, 2 h, 4 h, 8 h and 24 h. The cell lysates were probed for cyclin D1. The figure shows a representative blot. The lower band (36 KD) is cyclin D1 and the upper band is β-actin (43 KD).

The effects of auraptene on proliferation were evaluated in the human breast carcinoma cell lines, MDA-MB-231 and MCF-7. Proliferation was studied by measuring the formation of formazan product by living cells. Auraptene suppressed MDA-MB-231 cell proliferation by 50% at 12 μM and up to 85% at 25 μM (Figure 2-a). Increasing the concentration of auraptene to 50 μM did not achieve any higher suppression of cell proliferation. The effect was significant from 10 μM (p < 0.05). There was a reduction in the proliferation by about 23% at a concentration of 10 μM with no further change below 5 μM. The effect of auraptene on MCF-7 cells was not as prominent as in MDA-MB-231 cells. However, auraptene significantly reduced MCF-7 cell proliferation from 20–50 μM (p < 0.05). Auraptene suppressed MCF-7 cell proliferation by 26% at 20 μM and up to 49% at 50 μM (Figure 2-b). Based on the effects of auraptene on these human breast cancer cells, and on its previously demonstrated chemopreventive effects in other *in vivo *models, we hypothesized that auraptene would suppress MNU-induced rat mammary carcinogenesis.

### Auraptene reduced cyclin D1 expression in MCF-7 cells

Treatment of serum starved MCF-7 cells with IGF-1 induced a time-dependent increase in cyclin D1 expression which peaked at 8 h after IGF-1 addition to the medium. At 24 h after IGF-1 treatment, cyclin D1 expression was similar to that of control cells. Western blot analysis of MCF-7 human breast carcinoma cells in culture showed that 2 h pretreatment of auraptene (10 μM) resulted in nearly a total loss of IGF-1 induced cyclin D1 expression at 8 h (Figure 2-c).

### Dietary auraptene did not affect rat body weight

**Figure 3:**
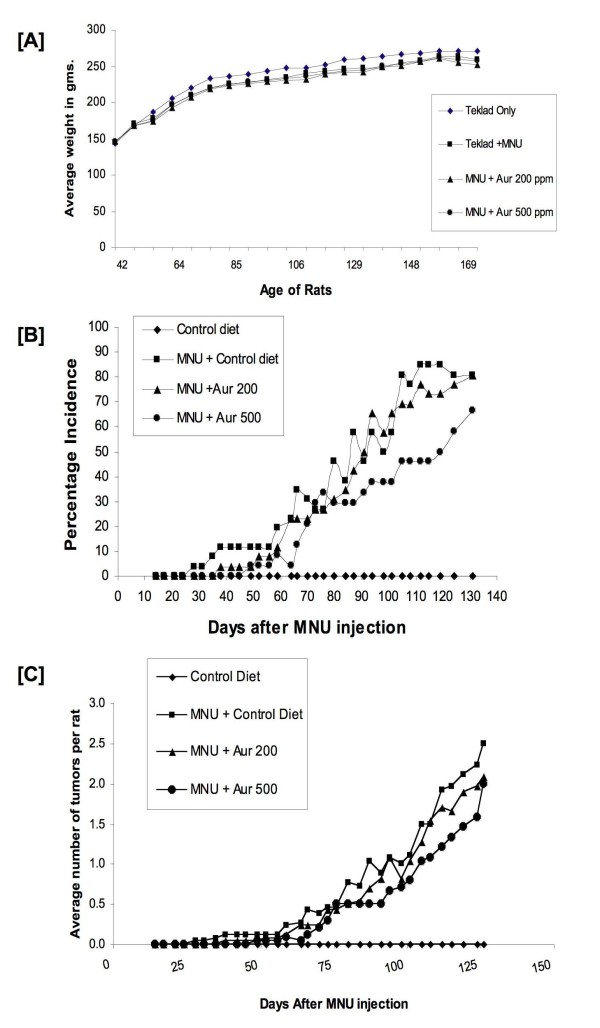
**Effects of dietary auraptene on mammary carcinogenesis**. **(A) **Rat body weight for the tumor study. Figures represent the means of each group. The data was analyzed by One-Way ANOVA followed by Tukey test. **(B) **Effects of dietary auraptene on rat mammary tumor incidence. Number of days after MNU administration is shown on the X-axis and the percentage of rats with tumors in each group is shown on the Y-axis. **(C) **Effects of dietary auraptene on tumor multiplicity. Number of days after MNU administration is shown on the X-axis and the average number of tumors per rat in each group is shown on the Y-axis.

Rat body weight was used as a gross indication of health and/or toxicity. After 2 weeks of MNU administration, the weight of the MNU treated rats was significantly lower than that of the control rats (Figure 3-a). This was observed until the end of the study. However, there was no significant difference in body weight between the groups treated with MNU (p < 0.05). Thus auraptene did not significantly affect body weight.

### Effects of auraptene on tumor incidence

In Figure 3-b, the percentage of tumor incidence (percentage of rats with tumor) in each group is shown. There were no palpable tumors in the control rats. The first palpable tumor in the MNU only group was seen at 28 days after MNU injection. In the MNU/Auraptene 200 ppm group, the first palpable tumor was seen at 38 days while it was seen at 49 days for MNU/Auraptene 500 ppm group after MNU injection, respectively. The effect of auraptene 500 ppm became evident after 77 days of MNU treatment. The tumor palpation data was analyzed every week to determine if auraptene had any effect on the tumor incidence. At 16 weeks, the maximum inhibitory effect of auraptene was observed (Figure 3-b and Table 1) where auraptene (500 ppm) significantly suppressed tumor incidence by 46%. Towards the end of the study the incidence in the MNU only group and the MNU/Auraptene 200 ppm group was 81% while that in MNU/Auraptene 500 ppm group was 67%. Thus, auraptene attenuated tumor incidence. The Cochran-Armitage trend test for binomial data showed that overall as the dose of auraptene was increased to 500 ppm there was a significant decrease in tumor incidence (p < 0.05).

**Table 1:** Summary of the effects of auraptene on rat mammary tumorigenesis

Group	Treatment	Incidence^a^	Multiplicity^a^	Median time to tumor (days)
*112 days (16 weeks)*				
2	MNU + Teklad (control)	84.6 ± 7.2	1.92 ± 0.27	
3	MNU + Aur. 200	76.9 ± 8.4	1.69 ± 0.27	
4	MNU + Aur. 500	45.8 ± 10.4^b^	1.21 ± 0.35^c^	
*126 days (18 weeks)*				
2	MNU + Teklad (control)	80.7 ± 7.8	2.50 ± 0.34	80
3	MNU + Aur. 200	80.7 ± 7.8	2.08 ± 0.33	92
4	MNU + Aur. 500	66.7 ± 9.8	2.00 ± 0.43	119^d^

^a ^Figures represent means ± SE. ^b^Statistically significant (p ≤ 0.05) using ANOVA followed by Tukey test. ^c^Statistically significant (p ≤ 0.05) using Mann-Whitney U-test. ^d^Statistically significant (p ≤ 0.05) using the log-rank test.

### Effects of dietary auraptene on tumor multiplicity and tumor burden

Next, we analyzed the effects of auraptene on tumor multiplicity (average number of tumors per rat). At 16 weeks, auraptene (500 ppm) significantly inhibited tumor multiplicity by 37% (Table 1). At the end of the study the average number of palpable tumors per rat in MNU only group was 2.5, while it was 2.08 in the MNU/Auraptene 200 group and 2.0 in the MNU/Auraptene 500 group (Figure 3-c). These results were not found to be significant. However, the total numbers of tumors were found to be significantly reduced in the MNU/Auraptene 500 group animals that had only 48 tumors when compared to MNU only group (p < 0.05) which had 65 tumors. The tumor burden (total tumor weights per rat and average tumor weight per rat in grams) was 6.62 ± 1.93 and 2.36 ± 0.77 in the MNU control; 6.52 ± 1.49 and 2.94 ± 0.79 in the MNU/Auraptene 200 ppm group; and 6.50 ± 2.10 and 1.79 ± 0.54 in the MNU/Auraptene 500 ppm group, respectively. These values were not significantly different from each other hence auraptene offered no significant protection in tumor burden at necropsy.

### Dietary auraptene delayed median time to tumor

**Figure 4:**
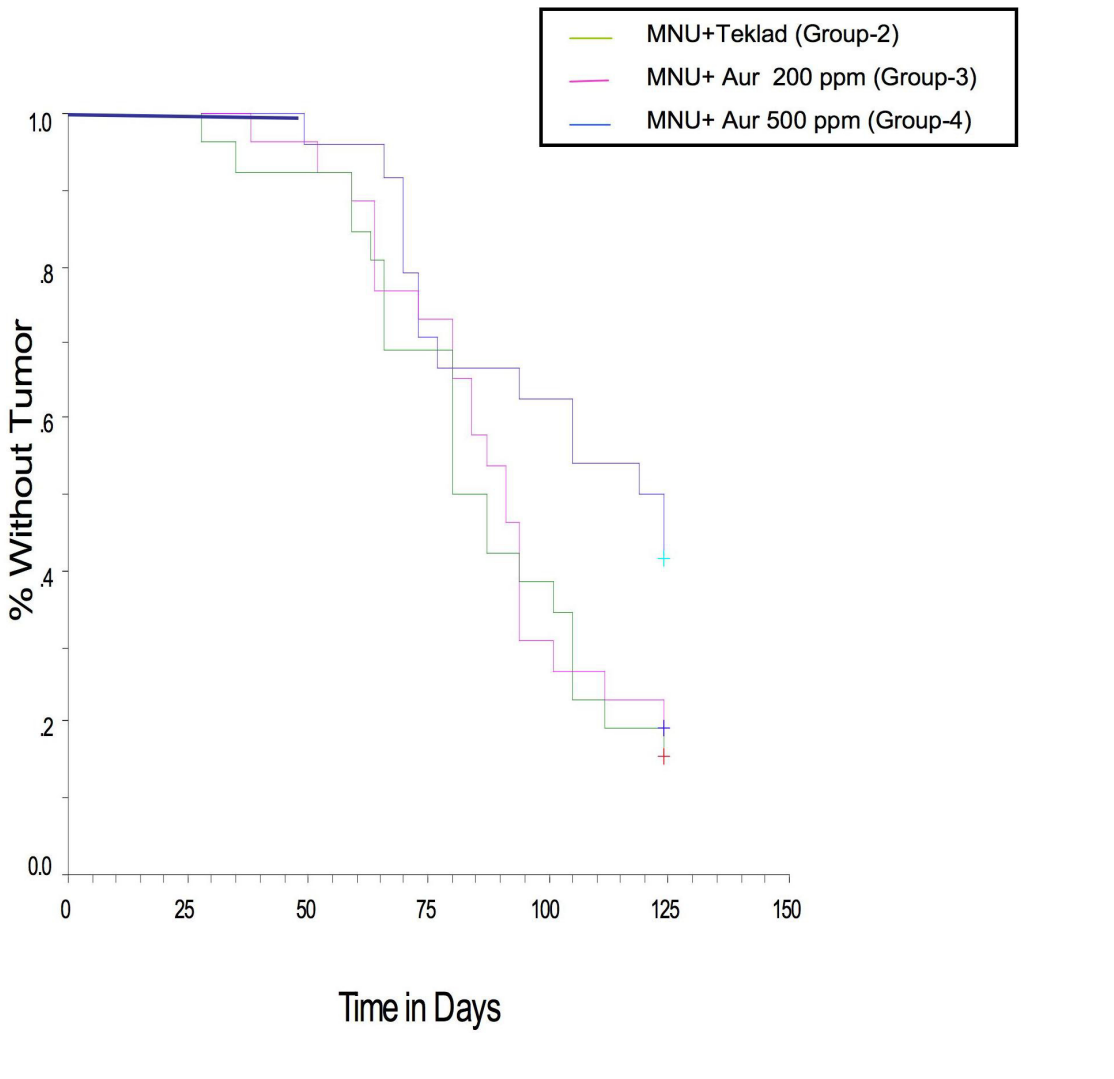
**Effects of dietary auraptene on median time to tumor**.

Median time to tumor is the time taken for the development of tumor in 50% of animals. The median time to tumor was found to be significantly delayed in the auraptene treated animals compared to MNU only group animals (Figure 4, log-rank test, p = 0.024). At the highest dose in this study (500 ppm) the median time to tumor was 119 days while that for the MNU only group (MNU) only treated group, it was 80 days (Table 1). Therefore, the delay in the 500 ppm group was 39 days (about 6 weeks). Thus auraptene significantly delayed the median time to tumor in the 500 ppm group. In the case of 200 ppm group the median time to tumor was 91 days and the delay was 11 days compared to the MNU group.

### Histopathology of mammary tumors

The H&E stained sections of the samples from this tumor study were analyzed for histopathology. Most tumors from the MNU treated groups were malignant adenocarcinoma of different types such as papillary, cribriform and comedo carcinoma. A few benign epithelial neoplasms such as lactating adenoma (adenomas with milk like substance in the lumen) and papillary adenoma were observed, in addition to the malignant tumors. In the Control group a papillary carcinoma was observed in the mammary fat pad on only one rat. This tumor was not found during palpation and necropsy. Some of the normal adjacent mammary glands in animals from all the groups showed exfoliation and/or mild hyperplasia. Overall, the histopathology of the tumor was consistent in all the MNU received animals irrespective of auraptene treatment; that is, in all groups most of the tumors were adenocarcinomas.

### Dietary auraptene suppressed cyclin D1 expression in rat mammary tumors

**Figure 5:**
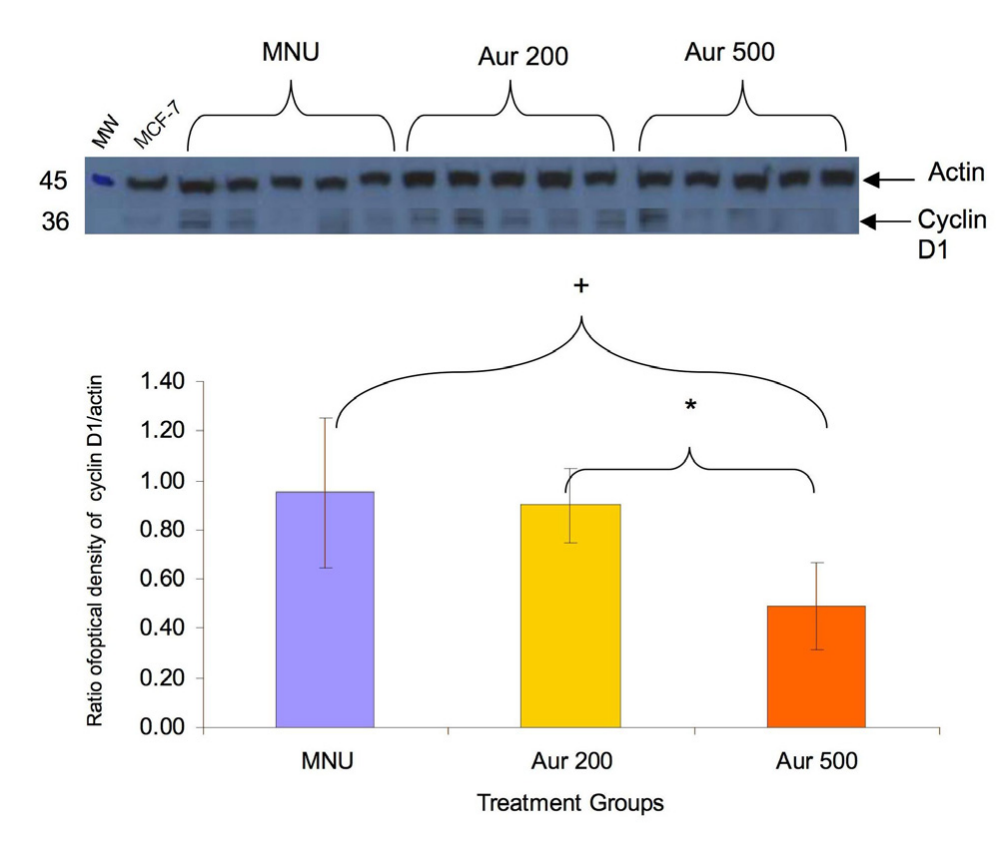
**Effects of dietary auraptene on cyclin D1 expression in rat mammary tumors**. From the same tumor study, rat mammary tumors were homogenized and the lysates were used for western blot to analyze the cyclin D1 expression. The top panel shows the representative blot. The bottom panel shows the graphical representation of the expression of cyclin D1. (+ statistically different from MNU group, * statistically different from Auraptene 200 group, p < 0.05, One-way ANOVA, Tukey test).

Based on the effect of auraptene on the expression of cyclin D1 expression in MCF-7 cells, the tumors from the rats were analyzed for cyclin D1 expression. Western blot analysis of rat mammary tumors (n = 10 per group) confirmed that auraptene (500 ppm) significantly reduced (p < 0.05) cyclin D1 expression by 49% compared to the MNU only group (Figure 5). The effect of 500 ppm dose was also significantly different than the 200 ppm dose of auraptene, indicating a dose-response. We also observed that MNU increased eIF4E protein expression in the tumors compared to control mammary gland. Although cyclin D1 is known to be translationally regulated by eIF4E and other pathways [27], there was no significant change in the eIF4E protein expression by auraptene (data not shown).

### HPLC analysis of auraptene in tissue

**Figure 6:**
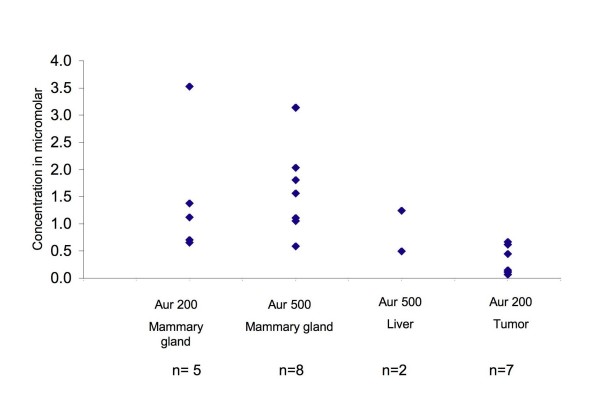
**Concentrations of auraptene in rat tissues from the tumor study**. The rat mammary glands, tumors and livers were weighed and homogenized. To the homogenate 80 μL of 100 μM curcumin was added as internal standard and extracted twice with ethyl acetate: acetone mixture (2:1 v/v). The extract was dried and resuspended in 1 mL 70% methanol/H_2_O and analyzed by HPLC (Shimadzu, SIL-10AF) with UV (254 nm) and fluorescence detection (Shimadzu, RF-535) at excitation and emission wavelengths of 329 nm and 390 nm respectively.

Since the ideal chemopreventive agent should be bioavailable after oral administration we tested whether auraptene reached the target tissue, the mammary glands. HPLC analysis with fluorescence detection indeed indicated that auraptene was reaching the mammary glands at both the doses of 200 and 500 ppm. Analysis of rat mammary glands indicated an average concentration of 1.4 ± 0.5 μM and 1.8 ± 0.3 μM for the auraptene 200 and 500 ppm groups respectively (means ± S.E.) (Figure 6). In addition to the mammary glands we analyzed the livers and mammary tumors for the presence of auraptene. Analysis of rat livers from auraptene 500 ppm group indicated an average concentration of 0.87 (mean, n = 2). The average concentration in mammary tumors from auraptene 200 ppm was 0.31 ± 0.98 μM (means ± S.E.). The tumors in the 500 ppm group were not available for analysis of auraptene concentration, as they were used in the western blot analysis of cyclin D1.

## Discussion

The current study was conducted to examine the effects of the citrus compound auraptene in the chemoprevention of breast cancer. Current prevention methods against breast cancer are limited by the number of compounds and their side effects. This warrants the need for new chemopreventive compounds against breast cancer. Even though auraptene has been shown to possess chemopreventive effects in many cancer models there is no data available regarding its effects on breast cancer, which is a major health threat to women. Overall, the results of the current study have demonstrated that citrus auraptene suppressed human breast carcinoma proliferation and IGF-1 induced protein expression of cyclin D1 *in vitro*. Furthermore, auraptene in the diet suppressed MNU-induced tumor incidence, number of tumors and significantly delayed median time to tumor in rats. Consistent with the cell culture results, auraptene also significantly decreased cyclin D1 in the rat mammary tumors. In chemopreventive studies the median time to tumor is a relevant parameter because it indicates the ability of a compound to prolong the onset of carcinogenesis.

Although auraptene at 500 ppm was found to be significant at 16 weeks after tumor MNU treatment in inhibiting tumor incidence and multiplicity, towards the end of the study both these effects were found to be not significant. This shows that auraptene's effect was mainly on delaying the onset of tumors rather than inhibiting the tumor formation at the highest dose in this study. In the future, analysis of rat tumors at different time points will give us more information regarding this effect. The effects of doses of auraptene higher than 500 ppm will also be worthwhile to study.

Chemoprevention of cancer requires a long-term administration of the compound through diet/oral administration. It is therefore required that the compound possesses little to no toxicity. Overall, auraptene was well tolerated by rats in the mammary carcinogenesis model as there was no observable change in the gross behavior of the rats and no significant weight loss among the rats treated with MNU ± auraptene compared to rats treated with MNU alone. Previous studies of auraptene have also not shown any toxic effect of this compound after dietary administration [13]. We have previously used auraptene in mice at up to 150–200 mg/kg bw [29] (and data not shown). In this study, our highest dose, 500 ppm of auraptene, corresponds to 10 mg of daily dose for 20 gm of average daily consumption of food by adult female rats. The average weight of the rats was around 250 gm and so they received approximately 40 mg/kg of auraptene per day in the 500 ppm group which much less than the safety limit.

As stated above, the basic idea behind chemoprevention of cancer with natural products is the administration of the natural compound in diet everyday. For an orally administered drug, bioavailability is an important factor, which contributes to its efficacy. Poor oral bioavailability can make natural compounds that are found to be effective in preventing carcinogenesis in some cancer models ineffective against carcinogenesis of internal organs. Quercetin is an example, which was found to be effective against colon carcinogenesis but was found to be ineffective in mammary carcinogenesis [25]. Coumarins are rapidly absorbed from the gastrointestinal tract and widely distributed [22]. Other coumarins like imperatorin, isoimperatorin and isopimpinellin have also been shown to be orally bioavailable [15,23,24,30]. Auraptene was detected in mammary glands, livers and tumors, suggesting that it is absorbed and distributed to the target organ. An article was recently published on the concentrations of auraptene in male Sprague-Dawley rats following oral administration [30]. From 1–4 h following gavage administration (500 μmol/kg bw, p.o.), auraptene was found in high concentrations in stomach and intestine (up to ~2000 nmol/g stomach, 100 nmol/g small intestine, and 20 nmol/g large intestine) [30]. Hepatic concentrations of auraptene reached up to ~25 μm within 1–4 h of oral administration, then dropped rapidly [30]. In our study, rats were fed the auraptene diet ad libitum, and were sacrificed during the day. Since rodents are typically nocturnal, we believe the reason the hepatic concentrations were lower in our study than in the Kuki et al study is because of the time differences. Furthermore, evidence suggests that glucuronidated and sulfated metabolites were found in serum and urine [30]. Taken together, these results suggest that auraptene possesses wide oral bioavailability.

The suppressive effects of auraptene on cyclin D1 expression were intriguing. Many oncogenic pathways can upregulate cyclin D1 and other proliferative, anti-apoptotic, metastatic and/or angiogenic proteins. A previous report demonstrated that in HT-29 human colorectal cancer cells in culture, auraptene (25 μM) suppressed the expression of c-Myc (which is often induced along with other pro-oncogenic proteins) [18]. The two major mechanisms for this effect were suppression of phosphorylated-ERK1/2 and eIF4B. The effect on c-Myc was at the translational level, as the levels of mRNA were unchanged, but the protein levels were decreased. Whether auraptene acts on ERK1/2 or eIF4B in the rat mammary model remains to be determined. However, our results suggest that auraptene may reduce cell proliferation through the suppression of cyclin D1 in MCF-7 cells and in the rat mammary tumors. Further experiments such as flow cytometry will be done to understand the effect on cyclin D1 on cell cycle arrest as reduction in cell proliferation may not be a direct effect of the suppression of cyclin D1 expression. To our knowledge, this is the first report of auraptene suppression of cyclin D1 protein expression *in vivo*. In a recent study in a Tissue Micro Array (TMA) format, we have shown that cyclin D1 protein is over expressed in the local breast cancer patients [27]. Thus suppression of cyclin D1 expression could play a major role in breast cancer prevention in the future.

In previous studies in other cancer models, auraptene has been shown to inhibit ODC [13,20], induce Phase-II enzymes including glutathione-S-transferase (GST) and quinone reductase (QR) [6], and suppress matrix metalloproteinases (MMPs) [19]. *In vitro*, auraptene also reduces superoxide generation in differentiated promyelocytic HL-60 cells [3], activates apoptotic pathways in Jurkat-T cells [4], suppresses MMPs in human colorectal adenocarcinoma cell line, HT-29 [18] and inhibits the expression of inducible nitric oxide and cyclooxygenase-2 in murine macrophage cell line RAW 264.7 [17,31]. Our research group reported that auraptene increased the activation of the antioxidant response element (ARE) in HepG2-ARE-GFP cells. Furthermore, the ability of orally administered auraptene (and other naturally occurring coumarins) to induce hepatic GST activities was significantly attenuated in Nrf2(-/-) mice [32]. This suggests that auraptene induces Phase II enzymes such as GST through an Nrf-2/ARE mechanism. Thus, auraptene does possess multiple mechanisms of chemoprevention.

In summary, dietary auraptene delayed MNU-induced median time to tumor and significantly suppressed cyclin D1 expression in the rat mammary tumors. The suppression of cyclin D1 was dose-dependent, with greater suppression at the higher dose of auraptene. This dose-dependent effect is consistent with that of the tumor latency. Overall, the *in vitro *and *in vivo *results both suggest that auraptene was effective in delaying the progression of tumor possibly through suppression of cyclin D1 protein expression.

## Conclusion

Overall, this study shows that auraptene significantly delayed the time to tumor and suppressed cyclin D1 which is a cell cycle protein that has been shown to play a major role in breast cancer. Auraptene reached its target organ, mammary glands, after dietary administration. The low/no toxicity of auraptene, its bioavailability in mammary glands after dietary administration and its chemopreventive effects makes auraptene an ideal compound to be further studied for clinical use. Further research needs to be done to understand its effects at different time points of cancer progression at higher doses. We are further studying the mechanism of regulation of cyclin D1 by auraptene and its downstream effects in human breast cancer cell lines.

## Competing interests

The authors declare that they have no competing interests.

## Authors' contributions

PK was responsible for conducting and interpreting the rat mammary tumor study and for writing the manuscript. KY and JT conducted the immunohistochemical studies and interpretation. DW did the *in vitro *study in MDA-MB-231 cells. JT, RG and BL analyzed eIF4E expression. MA assisted in the design and histopathological interpretation of the rat mammary tumor data. JM assisted in the design and statistical interpretation of the rat mammary tumor data. HK was responsible for the overall study in its design, experimentation, analysis and interpretation, and for editing the final version of the manuscript. All of the authors have seen the manuscript and agree to it.

## Pre-publication history

The pre-publication history for this paper can be accessed here:

http://www.biomedcentral.com/1471-2407/9/259/prepub
